# Supplementary material for: Low Expression of miR-375 and miR-190b Differentiates Grade 3 Patients with Endometrial Cancer
Source: Biomolecules. 2021 Feb 13;11(2):274. doi: 10.3390/biom11020274 (PMC7918779; doi:10.3390/biom11020274)

**Supplementary figure 1**. Exploratory analysis of the data. Principal component analysis reveals homogeneity in the miRNA profile of tested samples except sample from patient s46 (sample no. 24). Sample 24 was removed from the further analysis.

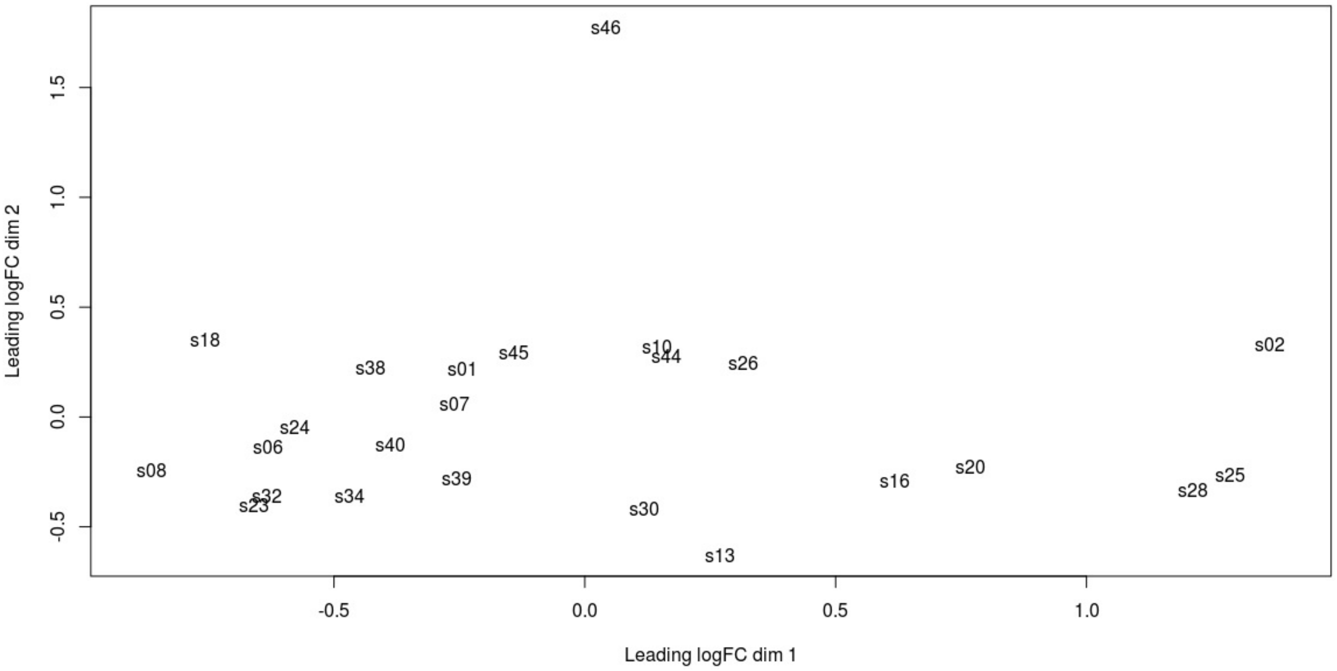

Supplement: Supplementary file 1 [file biomolecules-11-00274-s001.zip › biomolecules-1077888-supplementary/Supplementary_Figure_S1.pdf]
