# Supplementary material for: Low Expression of miR-375 and miR-190b Differentiates Grade 3 Patients with Endometrial Cancer
Source: Biomolecules. 2021 Feb 13;11(2):274. doi: 10.3390/biom11020274 (PMC7918779; doi:10.3390/biom11020274)
Supplement: Supplementary file 1 [file biomolecules-11-00274-s001.zip › biomolecules-1077888-supplementary/Supplementary_Table_S1.pdf]

Supplementary Table 1. Patients characteristic.

	**Patients phenotype**								**Tumour phenotype**			**Stage (FIGO)**
**No.**	**Patient ID**	**Age (years)**	**Height (cm)**	**Weight (kg)**	**Number of menstruations**	**Time to last menstruation (months)**	**Pregnancies**	**BMI**	**Tumour grade**	**Histological type according to WHO**	**Dualistic Bokhman classification**	
**1**	1	57	167	80	410	57	4	28,69	1	Endometroid carcinoma	1	IA
**2**	2	67	162	63	430	57	2	24,01	1	Endometroid carcinoma	1	IA
**3**	13	66	150	90	360	50	2	40,00	1	Endometroid carcinoma	1	IB
**4**	16	54	154	97	350	47	0	40,90	1	Endometroid carcinoma	1	IA
**5**	20	76	162	75	370	51	2	28,58	1	Endometroid carcinoma	1	IA
**6**	24	56	167	65	380	52	2	23,31	1	Endometroid carcinoma	1	IA
**7**	25	66	162	80	410	55	2	30,48	1	Endometroid carcinoma	1	IA
**8**	27	78	165	80	380	52	2	29,38	1	Endometroid carcinoma	1	IA
**9**	30	64	164	100	340	48	2	37,18	1	Endometroid carcinoma	1	IA
**10**	34	67	170	80	340	48	2	27,68	1	Endometroid carcinoma	1	IB
**11**	7	54	167	70	350	49	2	25,10	2	Endometroid carcinoma	1	IA
**12**	8	64	162	94	410	54	1	35,82	2	Endometroid carcinoma	1	IB
**13**	18	53	160	53	380	52	2	20,70	2	Endometroid carcinoma	1	IB
**14**	23	65	158	70	380	53	3	28,04	2	Endometroid carcinoma	1	IB
**15**	32	61	165	92	380	52	2	33,79	2	Endometroid carcinoma	1	IA
**16**	40	54	164	108	370	53	4	40,15	2	Endometroid carcinoma	1	IB
**17**	44	54	170	74	380	53	3	25,61	2	Endometroid carcinoma	1	IB
**18**	6	56	165	56	400	54	2	20,57	3	Endometroid carcinoma	2	IB
**19**	10	78	158	73	290	44	3	29,24	3	Clear cell adenocarcinoma	2	II
20	26	64	168	98	350	50	3	34,72	3	Undifferentiated carcinoma	2	IA
21	38	67	156	93	400	53	1	38,21	3	Endometroid carcinoma	2	II
22	39	77	165	67	320	45	1	24,61	3	Endometroid carcinoma	2	IA
23	45	46	158	52	310	46	3	20,83	3	Endometroid carcinoma	2	IA
24	46	58	165	66	430	57	2	24,24	3	Endometroid carcinoma/Undifferentiated carcinoma	2	IIIA
